# Shank2 Deletion in Parvalbumin Neurons Leads to Moderate Hyperactivity, Enhanced Self-Grooming and Suppressed Seizure Susceptibility in Mice

**DOI:** 10.3389/fnmol.2018.00209

**Published:** 2018-06-19

**Authors:** Seungjoon Lee, Eunee Lee, Ryunhee Kim, Jihye Kim, Suho Lee, Haram Park, Esther Yang, Hyun Kim, Eunjoon Kim

**Affiliations:** ^1^Department of Biological Sciences, Korea Advanced Institute for Science and Technology (KAIST), Daejeon, South Korea; ^2^Center for Synaptic Brain Dysfunctions, Institute for Basic Science (IBS), Daejeon, South Korea; ^3^Department of Anatomy, College of Medicine, Korea University, Seoul, South Korea

**Keywords:** autism spectrum disorder, Shank2, parvalbumin, GABAergic, social interaction, self-grooming, EEG, seizure

## Abstract

Shank2 is an abundant postsynaptic scaffolding protein implicated in neurodevelopmental and psychiatric disorders, including autism spectrum disorders (ASD). Deletion of *Shank2* in mice has been shown to induce social deficits, repetitive behaviors, and hyperactivity, but the identity of the cell types that contribute to these phenotypes has remained unclear. Here, we report a conditional mouse line with a *Shank2* deletion restricted to parvalbumin (PV)-positive neurons (*Pv-Cre;Shank2*^fl/fl^ mice). These mice display moderate hyperactivity in both novel and familiar environments and enhanced self-grooming in novel, but not familiar, environments. In contrast, they showed normal levels of social interaction, anxiety-like behavior, and learning and memory. Basal brain rhythms in *Pv-Cre;Shank2*^fl/fl^ mice, measured by electroencephalography, were normal, but susceptibility to pentylenetetrazole (PTZ)-induced seizures was decreased. These results suggest that *Shank2* deletion in PV-positive neurons leads to hyperactivity, enhanced self-grooming and suppressed brain excitation.

## Introduction

Shank family members are abundant postsynaptic scaffolding proteins that have been implicated in the regulation of synapse assembly and function (Sheng and Sala, 2001; Boeckers et al., 2002; Sheng and Hoogenraad, 2007; Grabrucker et al., 2011; Sheng and Kim, 2011; Jiang and Ehlers, 2013; Sala et al., 2015; Zhu et al., 2016; Monteiro and Feng, 2017).

Among the three known members of the Shank family (Du et al., 1998; Boeckers et al., 1999; Lim et al., 1999; Naisbitt et al., 1999; Sheng and Kim, 2000), Shank2 and Shank3 in particular have been strongly implicated in diverse neurodevelopmental and psychiatric disorders, including autism spectrum disorders (ASD), Phelan-McDermid syndrome and schizophrenia, and characterizations of animal models carrying Shank2/3 mutations have led to numerous suggested mechanisms to account for Shank-related pathologies, termed Shankopathies (termed as Shankopathies; Grabrucker et al., 2011; Jiang and Ehlers, 2013; Guilmatre et al., 2014; Sala et al., 2015; Schmeisser, 2015; Monteiro and Feng, 2017; Mossa et al., 2018).

More specifically, in humans, *SHANK2* (also known as ProSAP1) has been implicated in ASD, intellectual disability, developmental delay and schizophrenia (Pinto et al., 2010; Wischmeijer et al., 2011; Berkel et al., 2012; Leblond et al., 2012, 2014; Prasad et al., 2012; Rauch et al., 2012; Sanders et al., 2012; Chilian et al., 2013; Schluth-Bolard et al., 2013; Costas, 2015; Peykov et al., 2015a,b; Homann et al., 2016).

Several* Shank2*-deficient mouse lines, including those with germline deletion of exons 6–7, exon 7 or exon 24, have been developed. These models display diverse synaptic and behavioral abnormalities, and have provided insight into mechanisms that may underlie pathophysiologies specifically related to Shank2 (Berkel et al., 2012; Schmeisser et al., 2012; Won et al., 2012; Ey et al., 2013; Lim et al., 2017; Pappas et al., 2017).

Of the many possible approaches for understanding the normal and pathophysiological functions of Shank2, cell type-specific deletion of *Shank2* has demonstrated particular value, as shown by the synaptic and behavioral abnormalities observed in mice lacking Shank2 in excitatory neurons (Emx1-Cre and Camkii-Cre; Pappas et al., 2017; Kim et al., 2018), GABAergic neurons (Viaat-Cre; Kim et al., 2018), or Purkinje cells (Ha et al., 2016; Peter et al., 2016; Pappas et al., 2017).

In the present study, we found that Shank2 is expressed in GABAergic neurons, including parvalbumin (PV)-positive neurons, a neuronal cell type implicated in brain excitation and rhythms and brain dysfunctions, including ASD (Cardin et al., 2009; Gogolla et al., 2009, 2014; Sohal et al., 2009; Yizhar et al., 2011; Uhlhaas and Singer, 2012; Wöhr et al., 2015). We also generated and characterized mice with a* Shank2* deletion restricted to PV-positive neurons, and found that these mice displayed moderate hyperactivity and enhanced self-grooming. In addition, these mice showed suppressed susceptibility to seizures artificially induced by pentylenetetrazole (PTZ), suggestive of altered excitation in the brain.

## Materials and Methods

### Animals

To generate conditional *Shank2* knockout mice with exon 6–7 deletions in the PV-positive cell, *Shank2*^fl/fl^ mice in the C57BL/6J background (Ha et al., 2016) were crossed with *Shank2*^fl/fl^ mice that contain Pv-Cre transgene (Jackson Laboratory, #8069, 129P2/OlaHsd background). The Pv-Cre mice were back crossed to C57BL/6J mice for more than five generations. Resulting *Pv-Cre;Shank2*^fl/fl^ mice were used as a conditional knockout (cKO) group, and Cre-negative littermates (*Shank2*^fl/fl^ mice) were used as a control group (termed WT mice) in all experiments. Average ratios of WT and cKO mice, and males and females, per litter was both 182:181 and 182:181, respectively. Breeding was successful in >95% of cases, and *Pv-Cre;Shank2*^fl/fl^ mice showed survival rates and body weights that are comparable to those in WT mice. Mice were bred and maintained according to the Requirements of Animal Research at KAIST, and all experimental procedures were approved by the Committee of Animal Research at KAIST (KA2016-30). All mice were fed *ad libitum* and housed under 12-h light/dark cycle. Mice were kept in their home cages with siblings during behavioral test periods except for the three chamber test, direct social interaction test, and ultrasonic vocalizations test. To verify the genotype of mice, genomic PCR with three sets of oligonucleotide primers was used: for floxed (367 bp) and WT (253 bp) version of the Shank2 allele, forward, 5′-CGC ACT GTG GGC TCA TCA GAT G-3′, reverse, 5′-CAG ACG CAT CTT CCA GGG AAG C -3′; for Pv-Cre allele (650 bp), forward, 5′-GTG TTG CCG CGC CAT CTG C -3′, reverse, 5′-CAC CAT TGC CCC TGT TTC ACT ATC-3′; for WT Pv allele without Pv-Cre (500 bp), forward, 5′-CAG AGC AGG CAT GGT GAC TA -3′, reverse, 5′-AGT ACC AAG CAG GCA GGA GA -3′.

### Fluorescent *in Situ* Hybridization

In brief, frozen sections (14 μm thick) were cut coronally through the hippocampal formation. The sections were thaw-mounted onto Superfrost Plus Microscope Slides (Fisher Scientific #12-550-15). The sections were fixed in 4% formaldehyde for 10 min, dehydrated in increasing concentrations of ethanol for 5 min, and finally air-dried. Tissues were then pretreated for protease digestion for 10 min at room temperature. Probe hybridization and amplification were performed at 40°C using a HybEZ hybridization oven (Advanced Cell Diagnostics, Hayward, CA, USA). The probes used in this study were six synthetic oligonucleotides complementary to the nucleotide (nt) sequence 1675–2949 of Mm-Shank2, nt 62–3113 of Mm-Gad1-C3, nt 552–1506 of Mm-Gad2-C2, nt 464–1415 of Mm-Slc17a7/Vglut1-C2, nt 1986–2998 of Mm-Slc17a6/Vglut2-C3, and nt 2–885 of Mm-Pvalb-C2 (Advanced Cell Diagnostics, Hayward, CA, USA). The labeled probes were conjugated to Alexa Fluor 488, Atto 550, and Atto 647. The sections were hybridized with the labeled probe mixture at 40°C for 2 h per slide. Unbound hybridization probes were removed by washing the sections there times with 1× wash buffer at room temperature for 2 min. Following steps for signal amplification included incubations at 40°C with Amplifier 1-FL for 30 min, with Amplifier 2-FL for 15 min, with Amplifier 3-FL for 30 min, and with Amplifier 4 Alt B-FL for 15 min. Each amplifier solution was removed by washing with 1× wash buffer at room temperature for 2 min. The slides were viewed, analyzed, and photographed using TCS SP8 Dichroic/CS (Leica), and the ImageJ program (NIH) was used to analyze the images.

### Western Blot Analysis

Adult *Pv-Cre;Shank2*^fl/fl^ mice and control mice (3–4 months) were anesthetized with isofluorane and decapitated. The isolated brains were dissected on ice into the following regions: cortex, hippocampus, striatum and cerebellum. Each brain regions were immediately homogenized in ice-cold homogenization buffer (0.32 M sucrose, 10 mM HEPES, pH 7.4, 2 mM EDTA, 2 mM EGTA, protease inhibitors and phosphatase inhibitors). Samples on a nitrocellulose membrane were incubated with the following primary antibodies (Shank2, 1:1000, Synaptic Systems 162 202; α-tubulin, 1:1000, Sigma T9026) at 4°C, overnight. Immunoblot images were captured using the Odyssey Fc imaging system (LI-COR Biosciences).

### Immunohistochemistry

Adult mice were transcardially perfused with 0.9% saline and a low pH fixative solution (1% paraformaldehyde in 100 mM Na-acetate buffer, pH 6.0). After overnight postfixation, brains were carefully sectioned in 50–100 μm thickness with a vibratome. After 10-min washing in Tris-buffered saline (TBS) buffer, the brain sections were blocked in 2% of normal goat serum and 0.1% TritonX-100 in TBS for 2 h. Brain sections were incubated with diluted primary antibodies (Shank2, 1:500, Synaptic Systems 162 204 guinea pig; PV, 1:500, Swant *PV*27 Rabbit) in incubation buffer (2% of normal goat serum and 0.1% TritonX-100 in TBS buffer) at 4°C for 48 h and washed five times by TBS for 10 min. Brain sections were incubated with secondary antibodies conjugated with FITC or Alexa594 (Jackson ImmunoResearch) at 4°C for 48 h and washed five times by TBS buffer for 10 min. After mounting using Vectashield with DAPI, brain sections were imaged using a confocal microscope (10× and 63× objectives; LSM780; Carl Zeiss). For immunohistochemistry for PV-positive cells in Cg1 and M2 cortical regions, coronal brain sections (50 μm) from mice at the age of 16 weeks were incubated with primary antibodies (PV, Swant *PV*27 Rabbit; NeuN, MAB377, Millipore, Mouse) followed by secondary antibodies conjugated with Alexa 488 or Alexa 594 (Jackson ImmunoResearch). Confocal images of Cg1 and M2 cortical regions (800 μm × 1 mm) were acquired using a confocal microscope (10× objective; LSM780; Carl Zeiss) under the same condition (laser intensity, gain and pinhole size) across different mice and brain slices. A total of four slices were acquired from a single brain and subjected to image analyses using ImageJ software. For PV-positive cell counting, thresholded images were subjected to the counting of PV-positive and NeuN-positive cells. For the intensity of PV signals, the integrated intensity of PV signals in a particular cell was normalized to the cell area. All the cells in the 800 μm × 1 mm block were analyzed to determine PV/NeuN neuronal density and PV signals per neuron. Measurements from four slices were averaged to obtain *n* of 1.

### Mouse Neuron Culture

Cultured hippocampal neurons were prepared from embryonic day 17 fetal C57/BL6J mice. Briefly, dissected hippocampi were dissociated by incubating in papain for 15 min at 37°C, followed by gentle titration and plating on poly-D-lysine coated 18-mm glass coverslips with plating medium (Neurobasal-A medium supplemented with 2% B-27 plus, 2% fetal bovine serum, 1% GlutaMax and 1 mM sodium pyruvate, all from Thermo Fisher Scientific). Four hours after plating, all medium was replaced with fetal bovine serum-free culture medium (Neurobasal-A medium supplemented with 2% B-27 plus, 1% GlutaMax, and 1 mM sodium pyruvate) and then 50% replacement every 7 days.

### Immunocytochemistry

Days *in vitro* (DIV) 14 low density cultured mouse neurons (5 × 10^4^/18 mm round coverslip) were fixed in 4% paraformaldehyde/4% sucrose/Tyrode’s sol (136 mM NaCl, 2.5 mM KCl, 2 mM CaCl_2_, 1.3 mM MgCl_2_, 10 mM Na-HEPES, 10 mM D-glucose, pH 7.3) for 15 min, permeabilized for 5 min in 0.25% Triton X-100/Tyrode’s solution, and then incubated in 10% normal donkey serum for 30 min at 37°C for blocking. Cells were incubated with diluted primary antibodies (GAD67, 1:1000, Millipore MAB5406 mouse; PV, 1:1000, Millipore MAB1572 mouse; Shank2, 1:1000, Synaptic Systems 162 202 rabbit; MAP2, 1:1000, Synaptic Systems 188 004 guinea pig) in 3% NDS/Tyrode’s solution for 2 h at 37°C. Then, appropriate secondary antibodies (donkey-anti-mouse IgG-Alexa488, donkey-anti-rabbit IgG-Alexa594, or donkey-anti-guinea pig IgG-Alexa647, Jackson ImmunoResearch, 1:1000) were diluted in 3% NDS/Tyrode’s solution and applied for 45 min at 37°C. After mounting using Vectashield without DAPI, stained neurons were imaged using a confocal microscope (63× objective; LSM780; Carl Zeiss).

### Mouse Behaviors

We used male mice for all behavioral analyses. The behavioral assays were performed in the following order: Laboras test, open field test, repetitive behavioral test and/or hole-board test, three-chamber test and/or USV test, elevated plus-maze and/or light-dark test, and water maze and/or fear conditioning. Mouse behavioral experiments performed during the light-off periods.

### Three-Chamber Test

Social approach behavior was measured using three-chamber social-interaction tests (Silverman et al., 2010), as described previously (Ha et al., 2016); (Won et al., 2012; Ha et al., 2016; Kim et al., 2018). The three-chamber test was preceded by single-cage isolation for 3 days. This social isolation might increase social motivation in subject mice and mask social deficits, but we tried to match the protocol in the current study with those that we used in our previous studies. The three-chamber test consists of three 10-min sessions. After a 10-min habituation to the apparatus, a social target (age-matched 129 male mouse; stranger mouse 1/S1) and an inanimate object target (O) were introduced into wire cages on each side of the apparatus, and approach behaviors of the subject mouse were measured for 10 min. During the last 10 min, social novelty was evaluated by replacing the object target with a new social target (stranger mouse 2/S2). Sniffing time during each session was measured. The preference index was determined by calculating the numerical difference between the time spent sniffing S1 and O, or S2 and S1, divided by their sum × 100.

### Direct Social Interaction Test

Direct social interaction test was performed as described previously (Chung et al., 2015). The test was preceded by 3-day single-caged isolation. On the second day of isolation, each mouse was habituated in the gray square acryl box (33 × 33 × 22 cm) for 20 min under the light condition of ~25 lux. On the test day, without further habituation, an experimental male mouse was allowed to explore an age-matched novel 129/Sv male mouse for 10 min with video recording. Off-line video analysis was performed manually by an experienced researcher blind to the experimental condition. Specific sub-parameters of direct social interaction were nose-to-nose interaction, nose-to-tail (anogenital region) interaction, following and miscellaneous interactions such as body contacts and huddling from the experimental mouse to the novel social target.

### Ultrasonic Vocalizations

Ultrasonic vocalizations (USVs) during courtship behaviors were measured using unfamiliar female mice as strangers. Male adult mice were socially isolated by housing singly for 3 days; female adult mice were group-housed for the test. The test consisted of a 30-min habituation period followed by recording of USVs during courtship behaviors. A subject male mouse was placed in a novel test cage under a light condition of ~60 lux for 5 min to record its basal USVs without a female stranger/intruder. Next, a randomly chosen female stranger mouse was introduced into the cage and allowed to interact each other freely while recording courtship USVs of the subject mouse for 5 min. Avisoft SASLab Pro software was used to analyze USVs. Signals were filtered from 1 Hz to 100 kHz and digitized with a sampling frequency of 250 kHz, 16 bits per sample (Avisoft UltraSoundGate 116H). To generate spectrograms, the following parameters were used (FFT length: 256, frame size: 100, window: FlatTop, overlap: 75%), resulting in a frequency resolution of 977 Hz and a temporal resolution of 0.256 ms. Frequencies lower than 45 kHz were filtered out to reduce white background noises. The duration of direct social interactions in males towards females (not those in females towards males), defined by the total time spent in nose-to-nose social interaction, nose-to-tail social interaction, following and other social interactions such as body contacts, was measured manually in a blind manner by a trained experimenter. Social interactions during USV measurements were exclusively assessed in males. We did not determine whether measured USVs are from male or female mice because USVs under the context of male-female encounter, which likely represents courtship USVs, are mainly produced by males (Maggio and Whitney, 1985; Egnor and Seagraves, 2016). We did not measure female cycles assuming that group housing may synchronize the cycles.

### Repetitive Behaviors

Mice were placed in a new home cage containing bedding for 20 min. Durations of self-grooming, digging and jumping during the last 10 min of behavioral recordings were measured in a blinded manner by a trained experimenter. Self-grooming was defined as scratching or licking the face or body area. Digging was defined as acts of rooting out bedding using the head or forelimbs. Jumping was defined as leaping towards the wall or the lid of the cage using both hind limbs simultaneously, as described previously (Won et al., 2012).

### Hole-Board Test

The hole-board apparatus consists of a white acryl plate with 16 holes (3 cm diameter, aligned in a 4 × 4 format) and transparent side walls. Mice were allowed to freely explore the holes for 20 min. For correct observation of behaviors, video recordings were made from the bottom side. Head-bobbing counts towards the holes were measured manually by a trained experimenter in a blind manner. Center holes, 2 × 2 holes in the center of the box; corner holes, holes at the corner (total four holes); side holes, the rest of the holes.

### Open-Field Test

Mice were placed in the center of an acrylic open-field box (40 × 40 × 40 cm) and allow to freely explore the environment for 60 min under the light condition of 0 lux. Ethovision XT 13 software (Noldus) was used to determine offline the distant moved and time spent in the center zone, defined by a central area of 20 × 20 cm dimension. Mouse behaviors were recorded using a top-view infrared camera.

### Elevated Plus-Maze

The elevated plus-maze consist of two open arms (30 × 5 × 0.5 cm) and two closed arms (30 × 5 × 30 cm), and elevated to the height of 75 cm from floor. Elevated plus-maze apparatus was made by gray acryl. Light conditions around open and closed arms were ~300 and ~30 lux, respectively. In the test, mice were introduced to the center region of the elevated plus-maze and allow to explore freely for 12 min. All behaviors were recorded by a top-view infrared camera. Time spent in each arm was measured using Ethovision XT 13 software (Noldus).

### Light-Dark Test

The light-dark test apparatus consists of a light chamber (20 × 30 × 20 cm) and a dark chamber (20 × 13 × 20 cm), which is connected through an entrance between the two chambers (5 × 5 cm). The light conditions for light and dark chambers were ~300 and 0 lux, respectively. In the test, mice were introduced to the center of the light chamber and allowed to explore freely for 20 min. All behaviors were recorded using a top-view camera. Time spent in each chamber and frequency of transition between two chambers was measured using Ethovision XT 13 software (Noldus).

### Laboras Test

The Laboratory Animal Behavior Observation Registration and Analysis System (Laboras System Metris) was used to measure long-term movements of mice (locomotion, climbing, rearing, eating and drinking) in a home cage-like environment (Van de Weerd et al., 2001). Laboras monitoring was performed continuously for 96 h with a 12/12 light-dark cycle and *ad libitum* feeding. We did not validate the Laboras results by our own analyses, but previous studies have reported the results of Laboras validation (Van de Weerd et al., 2001; Quinn et al., 2003, 2006; Dere et al., 2015).

### Rotarod Test

Rotarod test was performed using a five-lane rotarod treadmill (Ugo Basile). Mice were trained for a total of six trials with three trials per day. Duration of each trial was 300 s, and the speed of treadmill was accelerated from 4 rpm to 40 rpm. Latency to fall was measured manually by a trained experimenter in a blind manner. Latency was regarded as 300 s when a mouse withstood full 300 s.

### Morris Water Maze

The maze was a circular water tank with the diameter of 120 cm. White water soluble non-toxic paint was dissolved into the water at ~20°C until the liquid became opaque enough to conceal the platform present under the water. All the walls of the test room were decorated with visually distinct signs to mark spatial locations. During the learning phase of the test performed for the first 5 days, the mice were subjected to three consecutive trials per day. In each trial, subject mice were placed in three different quadrants in a random manner. If a subject mouse fails to reach the hidden platform within the 1-min testing period, it was guided to the platform and taken out of the maze after a compulsory 15 s delay. On day 6 for the probe test, subject mice were placed in the center of the maze, and the movements were recorded for 1 min. On day 8, the platform position was moved to the opposite quadrant, followed reversal learning for 3 days and another probe test on day 11. Mouse swim paths and time spent in quadrant were analyzed using EthoVision 13 (Noldus).

### Fear Conditioning

Subject mice were placed in the fear chamber (Coulbourn Instruments) and allowed to freely explore for 10 min for habituation. On the next day, mice were allowed to explore the same chamber for 5 min, and five electrical foot shocks signed with tone were given to the subjects (2 s 0.7 mA, 1 min apart) during the last 5 min. After 24 h, mice were returned to the same chamber, and their freezing behaviors were recorded without foot shocks for 5 min. Freezing was defined as the absence of movement longer than 1 s and analyzed automatically using FreezeFrame (Coulbourn Instruments).

### EEG And PTZ-Induced Seizure

For EEG recordings, six small stainless steel screws (1 mm × 3 mm) were implanted on the skull (two for bilateral prefrontal, +1.8 mm AP, ±1.0 mm ML; two for bilateral parietal, −2.0 mm AP and ±1.8 mm ML; animal ground and reference screws were on the cerebellum). All screws were connected with a small header pin connector socket (Hirose Electric, H2021-ND) through soldered coated stainless steel wire and fixed on the skull using superglue (Loctite 401) and dental acryl. After 1 week of recovery period, EEG recordings were started. EEG recordings were performed using in a white acryl box (25 × 25 × 35 cm) with mice allowed to move around freely and a Cheetah Data Acquisition System (Neuralynx) with synchronized video recording. After 20-min habituation, EEGs were recorded for 40 min. After intraperitoneal injection of PTZ (Sigma; 40 mg/kg), additional EEGs were recorded for 20 min. EEG data were analyzed using a customized Matlab code. Baseline EEGs were analyzed using 5-min serial samplings, and the total power spectral density (PSD) was averaged per mouse by applying Fast Fourier Transform (FFT). Video recordings were used to analyze seizure stages defined as follows; stage 1, behavioral arrest; stage 2, myoclonic (jerk) seizures; stage 3: general tonic-clonic seizures, as described previously (Naydenov et al., 2014), stages of seizures that are optimal for the effect of a low-dose PTZ (Dhamne et al., 2017), which rarely elicits general tonic-clonic seizures. The seizure susceptibility score was defined as follows; 0.2 × (latency to stage 1) + 0.3 × (latency to stage 2) + 0.5 × (latency to stage 3).

### Statistics

Statistical details and results, as well as information on sex, age and number of mice used in this study, are described in Supplementary Table S1. For two-way ANOVA, multiple comparisons were not performed when interactions were not significant.

## Results

### *Shank2* Expression in Excitatory and Inhibitory Neurons

**Figure 1:**
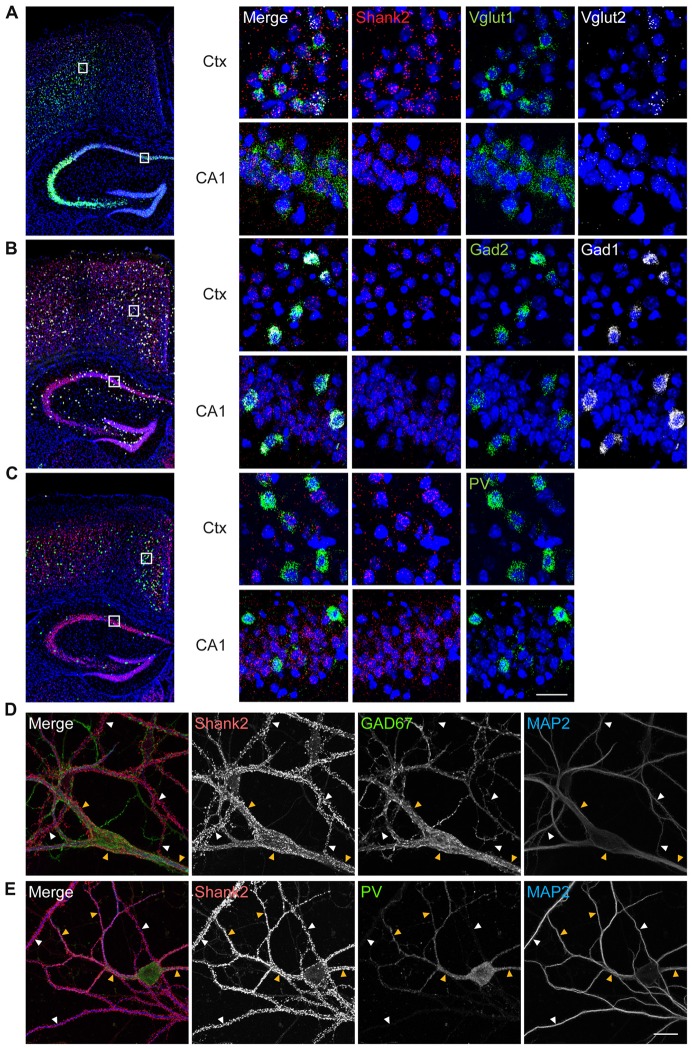
Shank2 is expressed in glutamatergic, GABAergic and parvalbumin (PV)-positive neurons in the brain. **(A–C)** Expression of Shank2 mRNA in glutamatergic **(A)**, GABAergic **(B)** and PV-positive **(C)** neurons in cortical and hippocampal regions, as determined by fluorescence *in situ* hybridization (FISH). Coronal sections from male mouse brains (8 weeks) were stained for Shank2 + Vglut1/2 (glutamatergic neuron markers), or Shank2 + Gad1/2 (GABAergic neuron markers), or Shank2 + Pvalb (PV-positive neurons), followed by counterstaining with the nuclear dye DAPI (shown in blue). A mixture of two probes (Vglut1 + Vglut2, or Gad1 + Gad2) was used to label all glutamatergic or GABAergic neurons. The indicated cortical and hippocampal regions in the image at left were enlarged (right panels) to highlight the details. Ctx, cortex; CA1, CA1 region of the hippocampus. Scale bar, 25 μm. **(D)** Detection of Shank2 protein signals on the dendrites (marked by MAP2) of GAD67-positive GABAergic neurons (yellow arrowheads) and GAD67-negative non-GABAergic neurons (white arrowheads) in dissociated mouse hippocampal cultured (days *in vitro* (DIV) 14) neurons. **(E)** Detection of Shank2 protein signals on the dendrites (marked by MAP2) of PV-positive GABAergic neurons (yellow arrowheads), PV-negative neurons (white arrowheads) in dissociated mouse hippocampal cultured neurons. Scale bar, 20 μm.

To determine which cell types in the mouse brain express Shank2, we performed fluorescence *in situ* hybridization experiments, using probes for Shank2 and markers of excitatory (Vglut1/2) and inhibitory (Gad1/2) neurons. Shank2 mRNA was detected in both Vglut1/2-positive glutamatergic neurons and Gad1/2-positive GABAergic neurons in multiple brain regions, including the cortex and hippocampus (Figures 1A,B). Shank2 mRNA signals were also detected in PV-positive GABAergic neurons in the cortex and hippocampus (Figure 1C). In cultured hippocampal neurons, Shank2 proteins were detected on the dendrites of GAD67-positive GABAergic neurons, GAD67-negative non-GABAergic neurons, and PV-positive GABAergic neurons (Figures 1D,E). These results suggest that Shank2 is expressed in both excitatory and inhibitory neurons.

### Generation and Characterization of *Pv-Cre;Shank2*^fl/fl^ Mice

**Figure 2:**
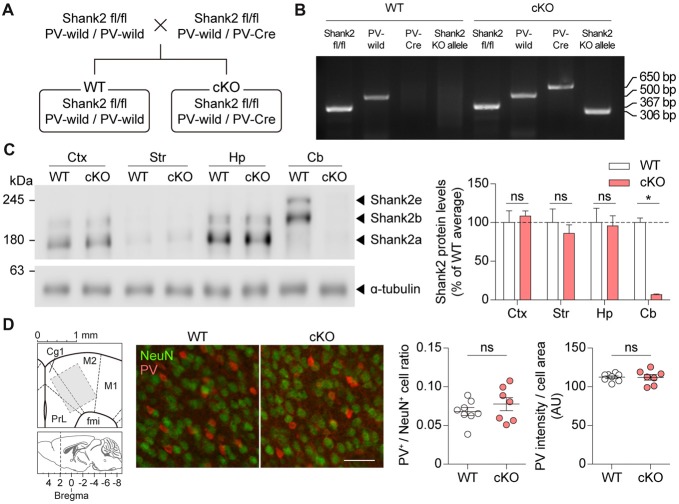
Generation and basic characterization of *Pv-Cre;Shank2*^fl/fl^ mice. **(A)** Breeding scheme for the production of WT and *Pv-Cre;Shank2*^fl/fl^ conditional KO (cKO) mice. Pv-Cre indicates mice heterozygous for the modified Pv allele, Pv-IRES-Cre; Pv-wild indicates normal WT mice without a Cre gene insert. **(B)** Genotyping of WT and *Pv-Cre;Shank2*^fl/fl^ mice, by PCRs on WT and cKO cerebellar samples (4 months). Note that only *Pv-Cre;Shank2*^fl/fl^ mice are positive for both the Pv-Cre allele and the Shank2 KO allele. **(C)** Levels of Shank2 protein in different brain regions of WT and *Pv-Cre;Shank2*^fl/fl^ mice (4 months). Ctx, cortex; Str, striatum; Hp, hippocampus; Cb, cerebellum. (*n* = 4 mice for WT and cKO, *P < 0.05, ns, not significant, Mann-Whitney U test). **(D)** *Pv-Cre;Shank2*^fl/fl^ mice (16 weeks) show normal numbers of PV-positive neurons and normal intensities of PV protein signals, as shown by the ratio of PV-positive cells and NeuN (neuronal marker)-positive cells and the intensity of PV protein signals normalized to cell area in Cg1 and M2 cortical regions. Note that a 200 × 200 μm block (middle) rather than the whole area (800 × 1000 μm; left) is shown to better visualize individual cells (*n* = 8 mice for WT and 7 mice for cKO, ns, not significant, Student’s *t*-test) The images from the Allen Mouse Brain Atlas was used in **(D)** (brain diagrams on the left; Lein et al., 2007). Scale bar, 50 μm.

To generate a mouse line that lacks Shank2 selectively in PV-positive neurons (*Pv-Cre;Shank2*^fl/fl^ mice), we crossed *Shank*2^fl/fl^ mice (floxed exons 6–7) with mice expressing Cre driven by the PV promoter (*Pv-Cre* mice) or with control mice (without Pv-Cre allele; Figures 2A,B). *Pv-Cre;Shank2*^fl/fl^ mice showed expected Mendelian ratios and normal levels of survival rates and body weights. Levels of Shank2 protein in the *Pv-Cre;Shank2*^fl/fl^ brain, revealed by immunoblot analysis, were substantially reduced, especially in the cerebellum, compared with *Shank2*^fl/fl^ mice (referred to hereafter as WT mice; Figure 2C), implying that Shank2 is normally strongly expressed in PV-positive Purkinje cells. Shank2 levels in other brain regions were comparable between genotypes, likely because PV-positive neurons are sparsely distributed in these regions. Numbers of Pv-positive neurons and levels of PV signals in each PV-positive neuron were normal in cortical regions of the *Pv-Cre;Shank2*^fl/fl^ brain (Figure 2D), dissimilar to the decreased levels of PV proteins in mice globally lacking Shank1 and Shank3 (Filice et al., 2016), although we have not tested global *Shank2* KO mice.

**Figure 3:**
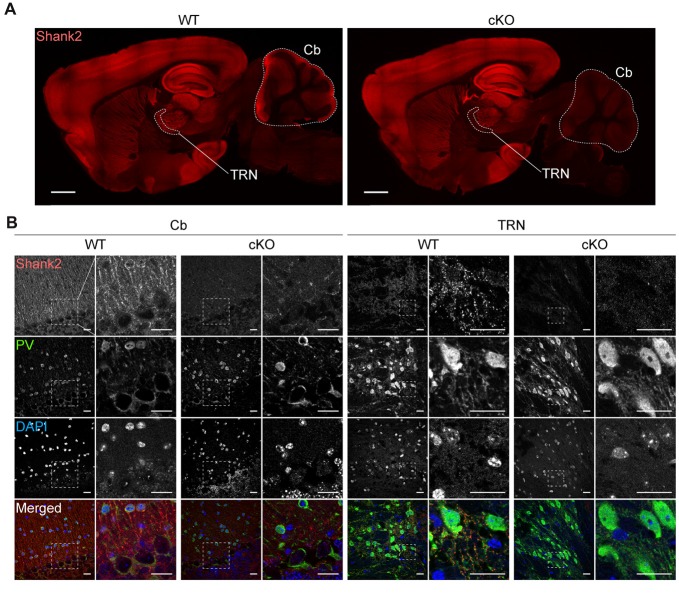
Shank2 protein levels are decreased in the thalamic reticular nucleus (TRN) and cerebellum in *Pv-Cre;Shank2*^fl/fl^ mice. **(A)** Decreased Shank2 protein levels in the TRN and cerebellum of *Pv-Cre;Shank2*^fl/fl^ mice (4 months), revealed by immunofluorescence staining of sagittal brain sections. Cb, cerebellum; TRN, thalamic reticular nucleus. Scale bar, 1 mm. **(B)** Reduced punctate Shank2 signals in dendrites and cell body regions around PV-positive cells in the TRN and cerebellum of *Pv-Cre;Shank2*^fl/fl^ mice. Scale bar, 20 μm.

Immunofluorescence staining showed a substantial decrease in Shank2 signals in the *Pv-Cre;Shank2*^fl/fl^ cerebellum (Figure 3A), results similar to those obtained by immunoblot analysis, and showed a similar decrease in the thalamic reticular nucleus (TRN) of the thalamus, which is densely populated by PV-positive neurons (Clemente-Perez et al., 2017). Enlarged images of these regions further revealed that neuropil areas in the cerebellum and TRN of *Pv-Cre;Shank2*^fl/fl^ mice were largely devoid of punctate Shank2 signals (Figure 3B); some weak signals detected in the cerebellum likely arose from non-PV neurons. The lack of a significant reduction in Shank2 signals in the cortex might be attributable to that ~6%–8% of cortical neurons are PV-positive neurons, an estimation based on that 15%–20% of cortical neurons are GABAergic neurons (Jones, 2009) and ~39% of cortical GABAergic neurons are PV-positive neurons (Gonchar et al., 2008). In addition, it might be because Shank2 is expressed in non-neural cells such as specific glial cell types, including ependymal cells, tanycytes, subpial/radial astrocytes, and choroid plexus epithelium (Redecker et al., 2001).

### *Pv-Cre;Shank2*^fl/fl^ Mice Show Moderate Hyperactivity but Normal Anxiety-Like Behavior

**Figure 4:**
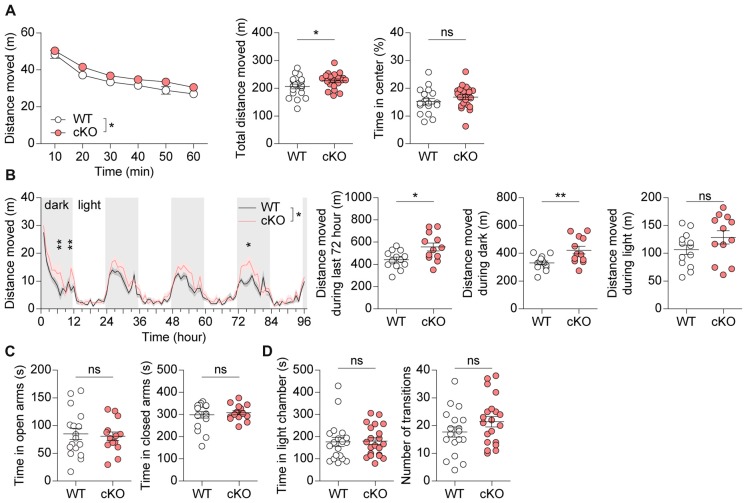
*Pv-Cre;Shank2*^fl/fl^ mice are moderately hyperactive, but show normal anxiety. **(A)** Moderate hyperactivity of *Pv-Cre;Shank2*^fl/fl^ mice (3–4 months) in the open field test, as measured by the total distance moved. Mean ± SEM (*n* = 19 mice for WT and *n* = 21 for cKO, *P < 0.05, ns, not significant, repeated measures of two-way ANOVA (for distance moved; the indicated significance is for main genotype effect [*P = 0.0483]; multiple comparisons at each time points were not performed because genotype × time interaction was insignificant [*P* = 0.8277]), and Student’s *t*-test (for total distance moved and time in center)). **(B)** Moderate hyperactivity of *Pv-Cre;Shank2*^fl/fl^ mice in the Laboras test, as measured by the distance moved during the last 72 h and during light-off periods. (*n* = 13 for WT and 12 for cKO, *P < 0.05, **P < 0.01, ns, not significant, repeated measures of two-way ANOVA with Bonferroni’s test (for distance moved), Student’s *t*-test (for distance moved during last 72 h and during light-on periods), and Welch’s *t*-test (for distance moved during light-off periods). **(C)** Normal anxiety-like behavior of *Pv-Cre;Shank2*^fl/fl^ mice (3–4 months) in the elevated plus maze test, as measured by time spent in open/closed arms. (*n* = 17 for WT and 14 for cKO, ns, not significant, Student’s *t*-test (for time in open arms) and Mann-Whitney U test (for time in closed arms)). **(D)** Normal anxiety-like behavior of *Pv-Cre;Shank2*^fl/fl^ mice (3–4 months) in the light-dark chamber test, as measured by time spent in the light/dark chamber. (*n* = 19 for WT and *n* = 21 for cKO, ns, not significant, Mann-Whitney U test (for time in light chamber), and Student’s *t*-test (for number of transition)).

In behavioral tests using male mice, *Pv-Cre;Shank2*^fl/fl^ mice displayed moderately enhanced locomotor activity in the open-field test (a novel environment) under complete darkness (0 lux) conditions compared with WT mice (Figure 4A). Similar hyperactivity was observed in the Laboras test (a familiar environment), in which mouse movements are monitored continuously for four consecutive days (96 h; Figure 4B). These results suggest that *Pv-Cre;Shank2*^fl/fl^ mice display moderate hyperactivity in both novel and familiar environments.

*Pv-Cre;Shank2*^fl/fl^ mice spent normal amounts of time in the center region of the open-field apparatus, open/closed arms of the elevated plus maze, and light chamber of the light-dark test apparatus (Figures 4A,C,D), suggesting that anxiety-like behaviors are normal in these mice.

### *Pv-Cre;Shank2*^fl/fl^ Mice Show Normal Social Interaction and USVs

**Figure 5:**
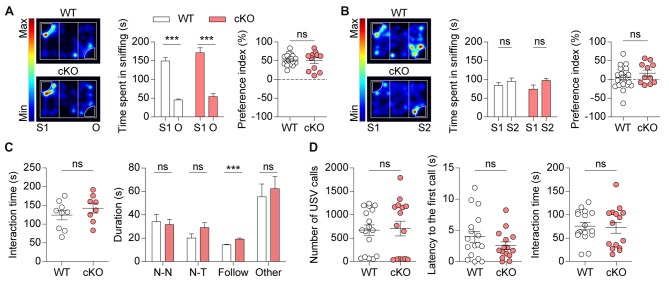
*Pv-Cre;Shank2*^fl/fl^ mice show normal social interaction and Ultrasonic vocalizations (USVs). **(A,B)** Normal levels of social approach in *Pv-Cre;Shank2*^fl/fl^ mice (3–4 months) in the three-chamber test, as measured by time spent sniffing a social stranger and preference index value (see “Materials and Methods” section). Note that these mice unexpectedly did not spend more time sniffing S2 relative to S1 under these conditions. (*n* = 19 for WT and *n* = 11 for cKO, ***P < 0.001, ns, not significant,paired Student’s *t*-test (for time spent sniffing of WT, cKO in S1–O session and WT of S1–S2 session), Wilcoxon matched-pairs signed rank test (for cKO of S1–S2 session), Welch’s *t*-test (for preference index in S1–O session), and Student’s *t*-test (for preference index in S1–S2 session). **(C)** Normal levels of direct social interaction in *Pv-Cre;Shank2*^fl/fl^ mice (3–4 months), as measured by total time spent in social interaction. Note, however, that these mice displayed an increase in time following a stranger, a sub-parameter of direct social interaction, but normal nose-to-nose (N-N) interaction, nose-to-tail (N-T) interaction, and other social interactions such as body contacts and huddling. (*n* = 9 for WT and *n* = 8 for cKO, ***P < 0.001, ns, not significant, Student’s *t*-test (for total time spent in direct social interaction, nose-to-tail interaction, following, other social interactions), and Mann-Whitney U test (for nose-to-nose interaction)). **(D)** Normal USVs in *Pv-Cre;Shank2*^fl/fl^ mice (3–4 months), as measured by the number of USVs and latency to first call. Note that direct male-female social interaction is normal in *Pv-Cre;Shank2*^fl/fl^ mice, as measured by the total time spent in direct social interaction (from males to females). (*n* = 17 for WT and *n* = 15 for cKO, ns, not significant, Mann-Whitney U test (for number of USVs), and Student’s *t*-test (for latency to first call and interaction time)).

We next tested social behaviors in *Pv-Cre;Shank2*^fl/fl^ mice. We found that these mice displayed normal social interactions in the three-chamber test, which measures social-approach behaviors (Silverman et al., 2010), as demonstrated by the time spent sniffing and the social preference index derived from sniffing time (Figure 5A; see figure legend for details). Social novelty recognition of an old vs. new social stranger could not be observed in WT or *Pv-Cre;Shank2*^fl/fl^ mice (Figure 5B) for unknown reasons.

In the direct social interaction test, *Pv-Cre;Shank2*^fl/fl^ mice showed normal levels of social interaction, as supported by total time spent in interaction (Figure 5C). Analysis of sub-parameters, however, revealed an increase in time spent in following a stranger, but normal time spent in nose-to-nose interaction, nose-to-tail interaction, or other interactions such as body contacts and huddling, suggesting that *Shank2* deletion in PV-positive cells affects a specific direct social interaction.

Male *Pv-Cre;Shank2*^fl/fl^ mice emitted normal levels of USVs towards a novel female mouse (courtship USVs), as measured by the number of USVs emitted and latency to the first call (Figure 5D). Direct social interactions observed during USV recordings were also comparable between genotypes. These results collectively suggest that social interaction and social communication are normal in *Pv-Cre;Shank2*^fl/fl^ mice.

### *Pv-Cre;Shank2*^fl/fl^ Mice Display Enhanced Self-Grooming

**Figure 6:**
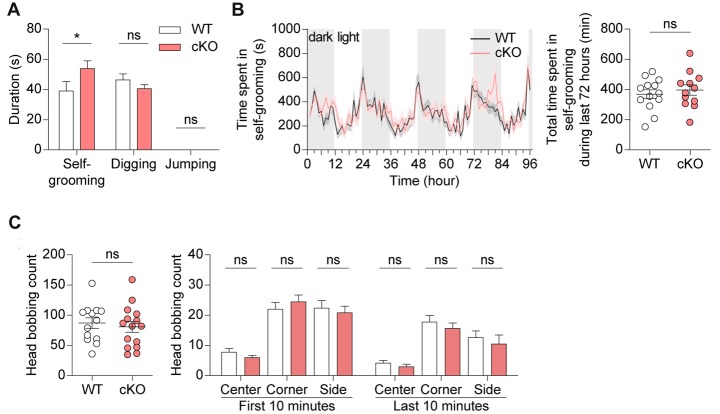
*Pv-Cre;Shank2*^fl/fl^ mice show enhanced self-grooming. **(A)** Enhanced self-grooming but normal digging and jumping in *Pv-Cre;Shank2*^fl/fl^ mice (3–4 months), as measured by the total time engaged in the indicated repetitive behaviors. (*n* = 16 for WT and *n* = 21 for cKO, *P < 0.05, ns, not significant, Mann-Whitney U test (for self-grooming), and Student’s *t*-test (for digging)). **(B)** Normal self-grooming in *Pv-Cre;Shank2*^fl/fl^ mice (3–4 months) in the Laboras test, as measured by the time spent self-grooming during the last 72 h. Note the occurrence of some episodes of enhanced self-grooming that did not affect the total self-grooming time. (*n* = 13 for WT and *n* = 12 for cKO, ns, not significant, repeated measures two-way ANOVA (for time spent self-grooming) and Student’s *t*-test (for total time spent self-grooming)). **(C)** Normal repetitive behavior in *Pv-Cre;Shank2*^fl/fl^ mice (3–4 months) in the hole-board test, measured by counting head bobs. (*n* = 13 for WT and *n* = 15 for cKO, ns, not significant, Student’s *t*-test (for total head bobbing count, head bobbing count on center and side during the first 10 min, and on corner during the last 10 min), and Mann-Whitney U test (for head bobbing count on corner during the first 10 min, and on center and side during the last 10 min)).

*Pv-Cre;Shank2*^fl/fl^ mice were next subjected to tests for repetitive behaviors. *Pv-Cre;Shank2*^fl/fl^ mice displayed enhanced self-grooming following introduction into a new home cage, while showing no changes in other repetitive behaviors (digging and jumping; Figure 6A). In contrast, these mice showed no overall increase in self-grooming in the Laboras test, measured as the total time spent self-grooming, especially during the last 72 h (i.e., after full habituation to the environment), despite some intermittent episodes of enhanced self-grooming (Figure 6B). In the hole-board test, another test for repetitive behavior (Takeda et al., 1998; Moy et al., 2008; Wang et al., 2011), *Pv-Cre;Shank2*^fl/fl^ mice showed largely normal levels of repetitive head bobbing (Figure 6C). These results collectively suggest that *Pv-Cre;Shank2*^fl/fl^ mice display novelty-induced enhanced self-grooming.

### *Pv-Cre;Shank2*^fl/fl^ Mice Show Normal Learning and Memory

**Figure 7:**
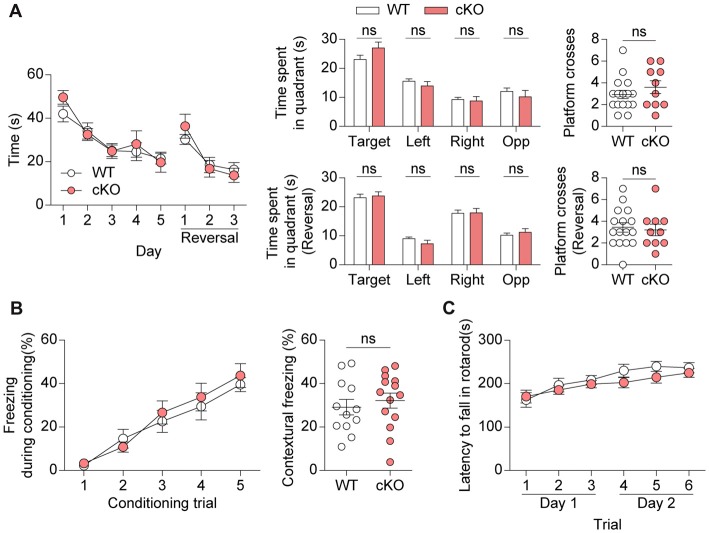
*Pv-Cre;Shank2*^fl/fl^ mice show normal learning and memory. **(A)** Normal learning and memory in *Pv-Cre;Shank2*^fl/fl^ mice (3–4 months) in standard and reversal variations of the Morris water maze test, as measured by the time to reach the platform, time spent per quadrant and number of platform crosses. (*n* = 16 for WT and *n* = 10 for cKO, ns, not significant, repeated measures of two-way ANOVA (for time to reach platform), Student’s *t*-test (for time spent in target, right, opposite quadrant during the probe test, right, opposite quadrant during the reversal probe test and platform crosses during the reversal probe test), and Mann-Whitney U test (for time spent in left quadrant during the probe test, target quadrant during the reversal probe test and platform crosses during the probe test), Welch’s *t*-test (for time spent in left quadrant during the reversal probe test)). **(B)** Normal fear learning and memory in *Pv-Cre;Shank2*^fl/fl^ mice (3–4 months) in the contextual test, as measured by levels of freezing during fear acquisition and 24-h fear retrieval (*n* = 12 for WT and *n* = 14 for cKO, ns, not significant, repeated measures of two-way ANOVA (for fear acquisition) and Student’s *t*-test (for contextual freezing)). **(C)** Normal motor coordination and learning in *Pv-Cre;Shank2*^fl/fl^ mice (3–4 months) in the rotarod test, as measured by the latency to fall. (*n* = 11 for WT and *n* = 16 for cKO, repeated measures of two-way ANOVA).

*Pv-Cre;Shank2*^fl/fl^ mice were then subjected to learning and memory tests. *Pv-Cre;Shank2*^fl/fl^ mice performed normally during both learning and probe phases of the Morris water maze test (Figure 7A). These mice also performed normally in the reversal phase of the Morris water maze test, in which the platform is moved to a new quadrant after initial learning. In the contextual fear-conditioning test, *Pv-Cre;Shank2*^fl/fl^ mice showed normal levels of fear acquisition and 24-h fear retrieval (Figure 7B), further suggesting that spatial learning and memory are normal in these mice. In addition, the rotarod performance of *Pv-Cre;Shank2*^fl/fl^ mice was comparable to that of WT mice (Figure 7C), suggesting that motor coordination and motor learning are not affected by conditional KO of *Shank2*. These results suggest that spatial and motor learning and memory are normal in *Pv-Cre;Shank2*^fl/fl^ mice.

### *Pv-Cre;Shank2*^fl/fl^ Mice Show Suppressed Susceptibility to Induced Seizures

**Figure 8:**
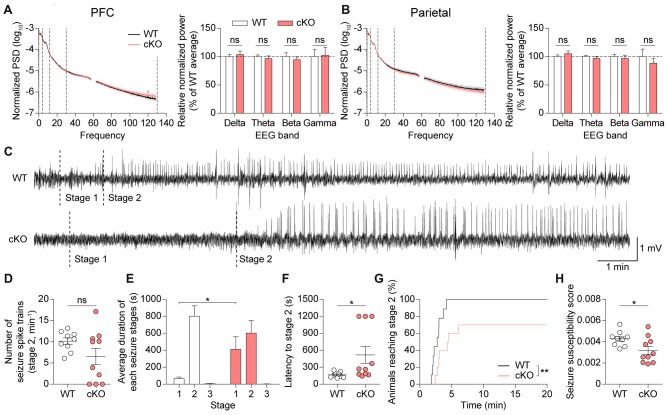
*Pv-Cre;Shank2*^fl/fl^ mice show suppressed susceptibility to induced seizures. **(A,B)** Normal basal EEGs in *Pv-Cre;Shank2*^fl/fl^ mice (3–4 months), as measured by total power spectral density (PSD) and EEG power in delta, theta, beta and gamma ranges. (*n* = 10 for WT and *n* = 10 for cKO, ns, not significant, Student’s *t*-test (for EEG power of delta, theta, beta ranges in mPFC, and EEG power of delta, theta, beta, gamma ranges in parietal), and Mann-Whitney U test (for EEG power of gamma range in mPFC)). **(C)** Sample EEG rhythms showing that pentylenetetrazole (PTZ)-induced seizures in *Pv-Cre;Shank2*^fl/fl^ mice start later than those in WT mice during a specific episode of behavioral seizures, suggestive of decreased susceptibility to PTZ-induced seizure. Stage 1, EEG traces associated with behavioral arrest; stage 2, EEG traces associated with myoclonic (jerk) seizure. **(D,E)** Suppressed susceptibility to PTZ-induced seizures in* Pv-Cre;Shank2*^fl/fl^ mice (3–4 months), as measured by the delay in the progression to subsequent seizure stages **(E)**. Note that the number of seizure spike trains are comparable between genotypes. (*n* = 9 for WT and *n* = 10 for cKO, *P < 0.05, ns, not significant, Welch’s *t*-test (for number of seizure spike trains) and Mann-Whitney U test (for duration in each seizure stages; i.e., stage 2 in WT vs. cKO)). **(F–H)** Suppressed susceptibility to PTZ-induced seizures in *Pv-Cre;Shank2*^fl/fl^ mice (3–4 months), determined by assessing behavior and measuring the latency to stage 2 seizure, cumulative percentage of animals reaching stage 2, and seizure susceptibility score (see “Materials and Methods” section for details). (*n* = 9 for WT and *n* = 10 for cKO, *P < 0.05, **P < 0.01, Mann-Whitney U test (for latency to stage 2), Student’s *t*-test (for seizure susceptibility score), and Log-rank test (for cumulative percentage of animals reaching stage 2)).

Because PV-positive neurons have a significant impact on the balance between excitation and inhibition in the brain and oscillatory rhythms under physiological and pathophysiological conditions (Cardin et al., 2009; Gogolla et al., 2009, 2014; Sohal et al., 2009; Yizhar et al., 2011; Uhlhaas and Singer, 2012; Wöhr et al., 2015), we performed electroencephalography (EEG) on *Pv-Cre;Shank2*^fl/fl^ mice. Mice were implanted bilaterally with a total of four electrodes on the surface of the skull in prefrontal and parietal regions. We found no significant differences in the spectral power of basal EEGs across delta (0.5–4 Hz), theta (4–12 Hz), beta (12–30 Hz), and gamma (30–130 Hz) frequency ranges in the prefrontal or parietal cortex (Figures 8A,B). These results are consistent with the lack of visible signs of spontaneous seizures in these mice.

Because *Pv-Cre;Shank2*^fl/fl^ mice could display altered responses to induced seizures, we tested their seizure responses to the γ-aminobutyric acid (GABA) antagonist PTZ (40 mg/kg). PTZ-induced seizure responses were assessed by simultaneously monitoring behavior and recording EEGs. EEG patterns after PTZ injection revealed a delay in the progression to subsequent seizure stages in *Pv-Cre;Shank2*^fl/fl^ mice (Figures 8C–E), suggesting that these mice are more resistant to PTZ-induced seizures. An analysis of seizure behaviors associated with epileptiform discharges in *Pv-Cre;Shank2*^fl/fl^ mice showed that the latency to the first myoclonic seizure (stage 2) was increased and the cumulative percentage of animals reaching stage 2 and the seizure susceptibility score were reduced, confirming that these mice are resistant to PTZ-induced seizures (Figures 8F–H). These results collectively suggest that brain rhythms are normal, but susceptibility to induced seizures is suppressed, in *Pv-Cre;Shank2*^fl/fl^ mice.

## Discussion

In the present study, we found that *Shank2* is expressed in GABAergic neurons including PV-positive neurons, in addition to glutamatergic neurons. We further found that a *Shank2* deletion (exons 6–7) restricted to PV-positive neurons leads to moderate hyperactivity, enhanced self-grooming, and suppressed susceptibility to induced seizures in mice.

Our finding that Shank2 is expressed in PV-positive neurons is in line with the previous demonstration that mRNAs for all three Shank isoforms (Shank1–3) are detectable in hippocampal PV-positive neurons by fluorescence labeling and laser-capture dissection followed by qPCR (Mao et al., 2015). Similarly, it has been shown that N-terminally EGFP-tagged Shank3 proteins are expressed in Gad2-positive GABAergic interneurons (Han et al., 2013). These results collectively suggest that Shank family proteins are widely expressed in GABAergic neurons, including PV-positive neurons.

Mice globally lacking Shank2 (exons 6–7) have previously been shown to display substantially increased (~2-fold) locomotor activity (Won et al., 2012). In the present study, we found that *Pv-Cre;Shank2*^fl/fl^ mice (exons 6–7) show relatively small increases in locomotor activity in both open-field (~10%) and Laboras (~25%) tests. These findings suggest that deletion of *Shank2* in PV-positive neurons contributes part of the hyperactivity observed in global *Shank2*-KO mice. Nonetheless, this contribution is still greater than that of Purkinje cells, a major type of PV-positive cell in the brain, as shown by the normal locomotor activity observed in two mouse lines lacking *Shank2* in Purkinje cells (exons 6–7 and exon 7; Ha et al., 2016; Peter et al., 2016). Therefore, the contribution of PV-positive neurons other than cerebellar Purkinje cells appears to be important for the hyperactivity observed in *Pv-Cre;Shank2*^fl/fl^ mice.

More recently, we reported behavioral phenotypes of mice with *Shank2* deletion (exons 6–7) restricted to CaMKII-positive excitatory neurons and Viaat-positive GABAergic neurons (Kim et al., 2018). Both mouse lines show hyperactivity phenotypes (~10–30%) that are milder than that in *Shank2* global KO mice (~2-fold). These results suggest that both excitatory and inhibitory neurons contribute to the hyperactivity in *Shank2* global KO mice and are in line with the moderate hyperactivity observed in *Pv-Cre;Shank2*^fl/fl^ mice in the present study. Given that the hyperactivity phenotype in *Viaat-Cre;Shank2*^fl/fl^ mice is apparently stronger than that in *Pv-Cre;Shank2*^fl/fl^ mice, non-PV GABAergic neurons might also contribute to the hyperactivity.

Notably, the hyperactivity phenotype in another *Shank2* mouse line that globally lacks exon 24 (corresponding to exon 15 in our nomenclature) is strongly recapitulated in mice in which the same *Shank2* deletion (exon 24) is restricted to Emx1-positive excitatory neurons, but not those in which the deletion is restricted to Pcp2-positive Purkinje cells or CaMKII-positive forebrain pyramidal neurons (Pappas et al., 2017). These observations suggest that a *Shank2* deletion (exon 24) in early excitatory (Emx1-positive) neurons but not late excitatory (CaMKII-positive) neurons is important for the development of hyperactivity in mice, although another *Shank2* deletion (exons 6–7) in late excitatory (CaMKII-positive) neurons has been shown to induce moderate hyperactivity (Kim et al., 2018).

*Pv-Cre;Shank2*^fl/fl^ mice (exons 6–7) in the present study display largely normal levels of three-chamber social-approach, direct social interaction, and courtship USVs, suggesting that deletion of *Shank2* in PV-positive neurons has minimal influences on social interaction and communication. These results sharply contrast with the relatively stronger social deficits observed in *Shank2* global KO mice, *CaMKII-Cre;Shank2*^fl/fl^ mice, and *Viaat-Cre;Shank2*^fl/fl^ mice (Won et al., 2012; Kim et al., 2018). This was also an unexpected finding given that PV-positive neurons have been strongly implicated the regulation of social functions in WT mice and mouse models of ASD (Gogolla et al., 2009, 2014; Yizhar et al., 2011; Sungur et al., 2014; Ito-Ishida et al., 2015; Vogt et al., 2015; Wöhr et al., 2015; Dong et al., 2016; Lauber et al., 2016; Filice and Schwaller, 2017; Kalbassi et al., 2017; Rapanelli et al., 2017; Selimbeyoglu et al., 2017; Cao et al., 2018; Filice et al., 2018; Tatsukawa et al., 2018). It remains to be determined whether a similar lack of social deficits would be observed in mice with other *Shank2* exon deletions, or *Shank3* deletions, restricted to PV-positive neurons.

*Pv-Cre;Shank2*^fl/fl^ mice (exons 6–7) displayed enhanced self-grooming in a new home cage, but not in a familiar environment, suggesting novelty-induced enhanced self-grooming. However, these mice showed normal levels of anxiety-like behaviors in elevated plus-maze, light-dark and open-field tests. In addition, these mice habituated normally in the open-field arena, and displayed normal levels of spatial learning and memory in Morris water-maze and contextual fear-conditioning tests. Therefore, it is unlikely that altered anxiety or cognitive function contributes to the novelty-induced, enhanced self-grooming in these mice. Notably, *Shank2* global KO mice (exon 6–7) and* CaMKII-Cre;Shank2*^fl/fl^ mice do not show enhanced self-grooming, whereas *Viaat-Cre;Shank2*^fl/fl^ mice do show strong enhanced self-grooming (Won et al., 2012; Kim et al., 2018). These results collectively suggest that GABAergic neurons may be more important than excitatory neurons for self-grooming, and that the enhanced self-grooming observed in* Viaat-Cre;Shank2*^fl/fl^ mice and *Pv-Cre;Shank2*^fl/fl^ mice is somehow masked by global *Shank2* KO.

The current study showed that *Shank2* deletion in PV-positive neurons does not affect other types of repetitive behaviors, such as digging and jumping, a finding that is in sharp contrast with the markedly suppressed digging and enhanced jumping observed in *Shank2* global KO (exons 6–7) mice (Won et al., 2012). This suggests that *Shank2* deletion in PV-positive neurons has little effect on repetitive behaviors apart from self-grooming. Previous studies on *Shank2* global KO (exon 6–7) mice as well as our current experiments on *Pv-Cre;Shank2*^fl/fl^ mice (exons 6–7) revealed normal head-bobbing repetitive behavior in the hole-board test. In contrast, mice with a* Shank2* deletion (exons 6–7) in Purkinje cells show enhanced repetitive head bobbing in this test. Therefore, *Shank2* deletion in Purkinje cells appears to be more closely related to the development of head-bobbing behavior than *Shank2* deletion in other PV-positive neurons.

Although additional details of repetitive behaviors in *Shank2*-mutant mice remain to be determined, our results are reminiscent of previous reports that deletion of ASD-risk genes in several types of GABAergic neurons, including Viaat-, Dlx1/2-, Dlx5/6- and PV-positive neurons, enhances repetitive behaviors. Specific examples include increased self-grooming in *Pv-Ctnnb1* mice (Dong et al., 2016), increased digging in *Pv-Grm5* mice (Barnes et al., 2015), increased head bobbing (hole-board test) in *Viaat-Mecp2* and *Dlx5/6-Mecp2* mice (Chao et al., 2010), and increased repetitive circling in *Dlx1/2-Scn1a* mice (Han et al., 2012).

*Pv-Cre;Shank2*^fl/fl^ mice displayed normal basal brain rhythms, but showed reduced susceptibility to PTZ-induced seizures, possibly reflecting changes in the functional properties of PV-positive neurons in these mice. These results are in line with previous reports suggesting that PV-positive neurons promote the generation and maintenance of seizure discharges and behaviors (Sessolo et al., 2015; Khoshkhoo et al., 2017). In addition, mice lacking the Shank2 relative, Shank3, have been shown to display enhanced EEG rhythms in the gamma range and markedly increased resistance to PTZ-induced seizures (Dhamne et al., 2017), results that are similar to our results in certain respects.

Shank2, which is mainly localized at excitatory synapses (Boeckers et al., 1999; Tao-Cheng et al., 2015; Heise et al., 2016), may regulate excitatory synapse formation and function in dendrites and consequently the output functions of PV-positive neurons, suggesting a potential mechanism for the resistance to PTZ-induced seizures in *Pv-Cre;Shank2*^fl/fl^ mice. Notably, excitatory transmission, neuronal firing, and inhibitory transmission to target neurons are reduced in PV-positive neurons in *Shank1* global KO mice (Mao et al., 2015). Although additional details of the roles of Shank2 in the regulation of brain rhythms and seizure susceptibility remain to be determined, our results suggest at minimum that Shank2 in mice is important for regulating brain excitation, a pathophysiology implicated in ASD in humans (Rubenstein and Merzenich, 2003; Gogolla et al., 2009; Cellot and Cherubini, 2014; Nelson and Valakh, 2015; Lee et al., 2017).

In conclusion, our results indicate that Shank2 is expressed in both glutamatergic neurons and GABAergic neurons, including PV-positive neurons, and suggest that Shank2 in PV-positive neurons is important for the regulation of locomotor activity, self-grooming and brain excitation.

## Author Contributions

SeungjoonL and EL generated mice and performed behavioral experiments. SeungjoonL, HP and EL performed EEG experiments. RK performed immunoblot experiments. JK, SeungjoonL and EL performed immunohistochemistry experiments. SuhoL performed dissociated neuron culture and immunocytochemistry. EY performed fluorescence *in situ* hybridization experiments. HK and EK wrote the manuscript.

## Conflict of Interest Statement

The authors declare that the research was conducted in the absence of any commercial or financial relationships that could be construed as a potential conflict of interest. The reviewer YB and the handling editor declared their shared affiliation.

## Supplementary Material

TABLE S1 Statistical details for all data presented in the figures. Different statistical methods were used based on the normality (checked by Shapiro-Wilk test) and variance of the data. Outliers were detected using the outlier test based on IQR method.
